# Accuracy of point-of-care testing devices for haemoglobin in the operating room: meta-analysis

**DOI:** 10.1093/bjsopen/zrad148

**Published:** 2024-01-24

**Authors:** Hilalion (San) Ahn, Tori Lenet, Richard W D Gilbert, Ranjeeta Mallick, Julie L V Shaw, Dean A Fergusson, Daniel I McIsaac, Guillaume Martel

**Affiliations:** Department of Surgery, The Ottawa Hospital, University of Ottawa, Ottawa, ON, Canada; Department of Surgery, The Ottawa Hospital, University of Ottawa, Ottawa, ON, Canada; Clinical Epidemiology Program, Ottawa Hospital Research Institute, Ottawa, ON, Canada; Department of Surgery, The Ottawa Hospital, University of Ottawa, Ottawa, ON, Canada; Clinical Epidemiology Program, Ottawa Hospital Research Institute, Ottawa, ON, Canada; Department of Pathology and Laboratory Medicine, University of Ottawa, Ottawa, ON, Canada; Clinical Epidemiology Program, Ottawa Hospital Research Institute, Ottawa, ON, Canada; Clinical Epidemiology Program, Ottawa Hospital Research Institute, Ottawa, ON, Canada; Department of Anesthesiology & Pain Medicine, The Ottawa Hospital, University of Ottawa, Ottawa, ON, Canada; Department of Surgery, The Ottawa Hospital, University of Ottawa, Ottawa, ON, Canada; Clinical Epidemiology Program, Ottawa Hospital Research Institute, Ottawa, ON, Canada

## Abstract

**Background:**

Point-of-care tests (POCT) for haemoglobin are increasingly used to guide intraoperative transfusion. However, their accuracy compared to central laboratory tests is unknown. The objective was to perform a systematic review and meta-analysis of method comparison studies assessing the accuracy of POCT *versus* central laboratory haemoglobin tests in patients undergoing surgery.

**Methods:**

Electronic databases were searched from inception to April 2020 (updated August 2023). Any methodological approach comparing haemoglobin measurements between POCT and central laboratory in patients undergoing surgery under anaesthesia in the operating room were included. Data abstraction was guided by PRISMA and risk of bias was assessed by QUADAS-2. Data were extracted independently and in duplicate by two reviewers. Outcomes included mean differences between POCT and central laboratory haemoglobin with associated standard deviations and 95% limits of agreement (LOA).

**Results:**

Of 3057 citations, 34 studies were included (*n* = 2427, 6857 paired measurements). Several devices were compared (pulse co-oximetry, *n* = 25; HemoCue, *n* = 10; iSTAT, *n* = 6; blood gas analysers, *n* = 10; haematology analyser, *n* = 2). Median sample size was 41 patients, and 11 studies were funded by device manufacturers. Fifteen of 34 studies had low risk of bias. Pooled mean differences (95% LOA) were: pulse co-oximeters 2.3 g/l (−25.2–29.8), HemoCue −0.3 g/l (−11.1–10.5), iSTAT −0.3 g/l (−8.4–7.8) and blood gas analysers −2.6 g/l (−17.8–12.7).

**Conclusion:**

All POCT examining intraoperative haemoglobin measurement yielded pooled mean difference LOAs larger than the allowable limit difference of ±4 g/dl. Intraoperative haemoglobin measured by POCT should not be considered interchangeable with central laboratory values and caution is necessary when using these tests to guide intraoperative transfusion.

## Teaser

Transfusions during surgery are commonly guided by point-of-care devices measuring haemoglobin values but their accuracy is unknown. This systematic review and meta-analysis of 32 studies shows that all point-of-care tests measuring intraoperative haemoglobin yielded pooled mean differences and limits of agreements beyond the allowable limit difference. Thus, haemoglobin measured by point-of-care devices during surgery should not be considered interchangeable with central laboratory values and caution is necessary when using these devices to guide intraoperative transfusions.

## Introduction

Red blood cell (RBC) transfusions are common in surgery and may account for 27–44% of all transfused RBC units in the hospital^1^. Transfusions can be life-saving, but also carry risks such as allergic and transfusion reactions, transfusion-associated acute lung injury and transfusion-associated circulatory overload, and have been associated with transfusion-related immunomodulation, which can potentially lead to worse perioperative and long-term oncologic outcomes in surgical patients^2–4^. Lastly, they are an expensive and limited resource, estimated to cost up to €696 per unit^5^.

It is well established that haemoglobin measurement plays a central role in any decision to transfuse RBCs. A recent systematic review of clinical practice guidelines providing transfusion recommendations revealed that of 10 guidelines, eight recommended transfusing based on haemoglobin values^6–9^. A 2016 Cochrane systematic review of studies guiding transfusions identified 31 trials that involved haemoglobin measurements as a trigger for transfusion^10^. Lastly, a recent survey of Canadian anaesthesiologists reported intraoperative haemoglobin levels to be the most important parameter for transfusion decision-making—more important than blood loss or haemodynamics^11^.

Haemoglobin can be assessed by several methods. Considered to be the gold standard and part of the complete blood count, the haemoglobincyanide (HiCN) method uses an internationally accepted reference calibrator and provides a measured haemoglobin concentration^12^. However, this process is time-consuming and increasingly less useful to guide intraoperative transfusion in the context of acute bleeding. More recently, point-of-care testing devices for haemoglobin (POCT-Hb) have evolved and become the current standard of care during surgery. These devices are relatively simple to use and yield results within seconds to minutes, leading to greater clinical use in the operating room.

There are several classes of POCT-Hb. The first type chemically converts haemoglobin found in the blood sample to azide-haemoglobin, which is then measured by absorption photometry. This technology can yield a haemoglobin value from 10 µl of whole blood in 10–60 s^13^. A commonly used device in this category is HemoCue (HemoCue AB, Angelholm, Sweden). A second method provides a calculated haemoglobin value based on the conductometric method. Using 65–100 µl of whole blood, it calculates haemoglobin in 120 s by multiplying the haematocrit (hct) by a proportionality constant (Hb (g/l) = hct (%) × 3.4)^14^. A commonly used device in this category is iSTAT (Abbott Laboratories, Abbott Park, IL, USA). A third common method is pulse oximetry. This technology uses 12 or more wavelengths of light to measure total haemoglobin, and allows for non-invasive continuous monitoring^15^. Devices in this category include Masimo Radical-7 (Masimo Corp., Irvine, CA, USA).

POCT-Hb devices have been validated with static haemoglobin values, such as in healthy blood donors^16–18^, or in non-operative settings, such as the intensive care unit or emergency room^19–21^. The use of POCT-Hb in the intraoperative setting, where haemoglobin levels can change quickly due to bleeding and rapid fluid shifts from concurrent intravenous fluid administration^22^, is relatively untested. There are also few studies assessing POCT-Hb devices within the critical zone of potential transfusion of 60–100 g/l, highlighting a major criticism of existing evaluations^23–25^. The relationship between POCT-Hb and transfusion decisions has been emphasized by multiple other authors^23,26–30^.

Despite the lack of evidence validating their use in the operative setting, POCT-Hb devices have become ubiquitous to guide intraoperative transfusion decisions. The aim of this study is to perform a systematic review and meta-analysis of method comparison studies assessing the accuracy of POCT-Hb compared to central laboratory testing in patients undergoing surgery.

## Methods

### Information sources

This study was registered with PROSPERO (CRD42021233103). Reporting of this review was guided by the PRISMA statement (*Table S1*).

A systematic search was designed by an information specialist (R.S.). The search included EMBASE (1947 to August 2023), Ovid MEDLINE (1946 to August 2023) and EBM Reviews—Cochrane Central Register of Controlled Trials (August 2023).

### Search strategy

The full electronic search strategy was peer reviewed and conducted following the Peer Review of Electronic Search Strategies (PRESS) guidelines^31^. The search was not limited by language or patient population. Grey literature was included in the form of conference abstracts. References of included articles were reviewed manually for other relevant studies. Finally, the list of included references was circulated to a small group of experts in anaesthesiology and transfusion medicine to identify any additional missing studies. The search strategy is reported in the *Supplementary Methods*.

### Eligibility criteria

Study participants were required to be patients undergoing any surgery under general or neuraxial anaesthesia in an operating room. Studies that included data from other clinical settings were also included if data provided for the intraoperative period were reported separately. Studies of interest were those that compared haemoglobin values provided by POCT and the reference central laboratory standard. The gold standard was defined as the HiCN test method with blood samples collected in ethylenediaminetetraacetic acid (EDTA) vacuum collection tubes and processed by a haematology analyser in a central laboratory. POCT devices included any non-invasive measurements via pulse co-oximetry, via occlusion spectroscopy and via transcutaneous reflection. This review also included invasive measurements via absorption photometry, calculations via conductivity or via blood gas analysers. Sampling between the POCT device and central laboratory must have been taken concurrently or analysed from the same sample.

Studies that did not account for within-individual correlation between successive measurements (that is repeated haemoglobin measurements taken from the same patient but analysed as separate data points) were eligible for inclusion, but were accounted for in the analysis. Given differences in transfusion practice, neonatal populations were excluded. Studies that assessed the accuracy of POCT-Hb *versus* central lab haemoglobin in settings outside the operating room were excluded, such as endoscopy or minor bedside procedures. Finally, studies in which only the haemoglobin mean difference was provided, without the standard deviation (s.d.) or 95% limits of agreement (LOA), were excluded if those values could not be provided by the authors or derived from other data points or graphs.

### Effect measures

The primary outcome was the bias or mean difference (MD) between POCT-Hb minus central lab haemoglobin measurements and its s.d. If not provided in the text, 95% LOA intervals were also calculated using the following formula: 95% LOA = mean difference ± 1.96 × s.d.

### Data collection process

**Fig. 1:**
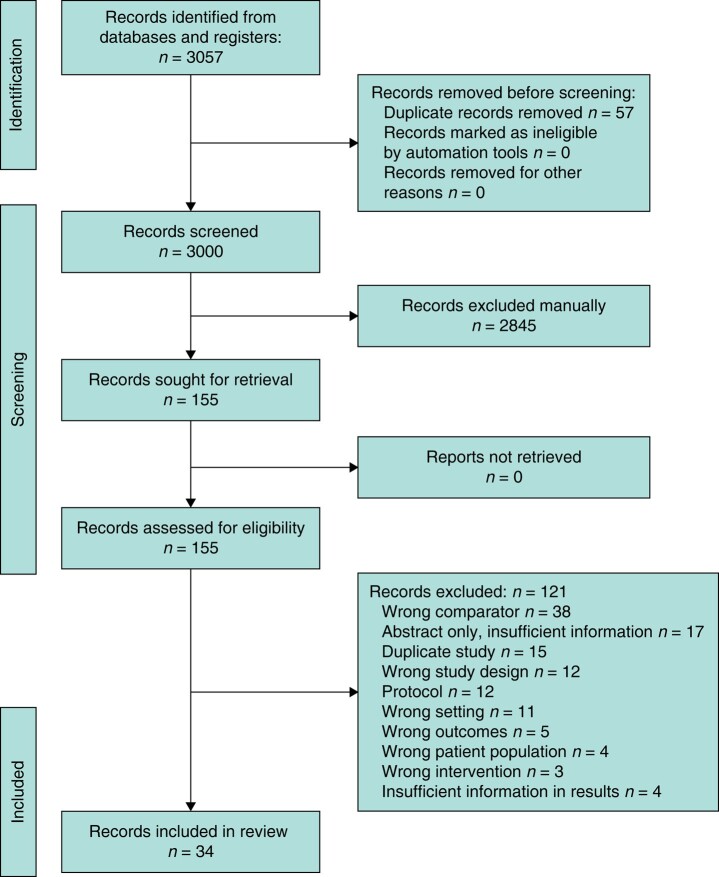
PRISMA flow diagram

Articles identified through the search strategy were imported into Covidence (Covidence, Melbourne, Australia), an online citation manager for systematic reviews^32^. Title, abstract and full-text screening were performed independently and in duplicate by two reviewers (H.A., T.L.). Authors were contacted for any uncertainties regarding eligibility. At both stages of review, any discrepancies were documented, discussed and adjudicated by the senior author (G.M.). Google Translate was used to translate non-English or non-French articles. Reasons for exclusion were documented and reported in the PRISMA flow diagram (*Fig. 1*).

### Data items

A standard data extraction form was created using Covidence, which was then exported into a Microsoft Excel spreadsheet (Microsoft Corporation, Redmond, WA, USA). Information gathered comprised study characteristics such as year of publication, location, funding source, sample size and study design and patient characteristics including age, sex and weight. Operative characteristics including type of surgery, anaesthesia, duration, blood loss, transfusions, fluid balance and operative interventions were also recorded. Finally, POCT device, central lab analyser, number of paired measurements, timing of samples and sampling site (arterial, venous or capillary) were also documented. Data extraction was performed independently by two reviewers (H.A., T.L.) and authors were contacted for any uncertainties.

### Statistical analysis

The MD, 95% LOA and s.d. were extracted from each study. Confidence bands of LOA were estimated using a random effects model and robust variance estimation (RVE), as previously described by Williamson *et al*.^33^. For studies that did not account for within-individual correlation for repeated measurements, the s.d. was adjusted to provide an RVE. Confidence bands of LOA are presented by the lower 95% limit for the lower value of the LOA and the upper 95% limit for the upper value of the LOA. The pooled estimate of MD, 95% LOA and confidence bands of LOA were estimated using the approach described by Tipton and Shuster^34^. The correlation coefficient (*r*) and kappa coefficient (κ) were also extracted. Heterogeneity was determined by the Chi square test (significance level 0.05) combined with the *I*^2^ statistic. Possible sources of heterogeneity were investigated with predefined subgroup analyses including funding source, blood loss, haemoglobin range and sampling site. A sensitivity analysis was performed based on risk of bias. Meta-analysis of each outcome was performed using R (version 4.0.2) in R Studio (version 2022.07.2 build 576), using the base and stats packages^35^.

### Study risk of bias assessment

The quality of included studies was assessed according to the revised Quality Assessment of Diagnostic Studies (QUADAS-2) guidelines^36^. This tool consists of four domains including patient selection, index test, reference standard, and flow and timing. The risk of bias is assessed for each domain in addition to applicability for the first three domains. The questionnaire was adopted from Kim *et al.*^37^ and tailored to this review, reported in the *Supplementary Methods*. If answers to all signalling questions for a domain were ‘yes’, then risk of bias for that domain was judged low. If two or more domains were deemed ‘high’ risk of bias, then the overall assessment for the study was judged ‘high’ risk of bias. Quality assessment was performed by three reviewers (H.A., T.L., R.G.) independently and any disagreements were discussed and adjudicated by the senior author (G.M.).

## Results

### Extent of evidence identified

Results from the search strategy are shown in *Fig. 1*. A total of 3000 de-duplicated citations were identified for title and abstract review. A total of 155 citations were eligible for full-text review, of which 34 studies were included in the systematic review.

### Characteristics of included studies

Study characteristics are reported in *Table S2*. Among the included studies, 25 compared pulse co-oximetry devices to central lab (*n* = 1110 patients, 4059 paired measurements)^26–28,38–58^, nine compared HemoCue (*n* = 525 patients, 1962 paired measurements)^26,27,39,44,57,59–63^, six compared iSTAT (*n* = 146 patients, 294 paired measurements)^19,28,40,47,59,64^ and 10 compared blood gas analysers (*n* = 821 patients, 3381 paired measurements)^26,28,39,41,49,50,57,60,63,65^. Two studies compared different POCT haematology analysers (*n* = 295 patients, 553 paired measurements)^66,67^ and were not meta-analysed. There were 27 full-text papers and seven conference abstracts. Median publication date was 2014 (range 1991–2023). Median sample size was 41 (range 6–348). Eleven studies were funded by device manufacturers, one was funded by the U.S. military, three were university-funded, 10 were not funded and 10 did not report funding sources. Participants in 25 studies received a general anaesthetic, one under spinal, and eight were not reported. Eight studies included cardiac surgery, seven neurosurgery, two transplant, two orthopaedic surgery, two gynaecological surgery, two urology, one vascular and 12 were not specified. Nine studies quantified intraoperative blood loss (mean range 100–3400 ml) and three reported blood transfusions (mean range 0–750 ml). Three studies reported mean difference of POCT-Hb devices within the critical transfusion zone of 60–100 g/l.

All included studies reported haemoglobin MD, 28 reported 95% LOA and 17 reported s.d. of the MD. Six studies accounted for within-individual correlation between successive measurements^26,27,40,52,53,65^.

### Risk of bias assessment

The methodological quality of included studies is shown in *Table 1*, based on the revised QUADAS-2 guidelines. In total, 15 of 34 studies were assessed to have low risk of bias. Patient selection and flow and timing were assessed to have the highest risk of bias across all studies, while index test and reference standard had the lowest risk. Only six studies reported a sampling method (that is, random, consecutive or convenience sample) and only four papers reported on their types of study design (that is, superiority, equivalence, inferiority).

**Table 1:** QUADAS-2 risk of bias assessment

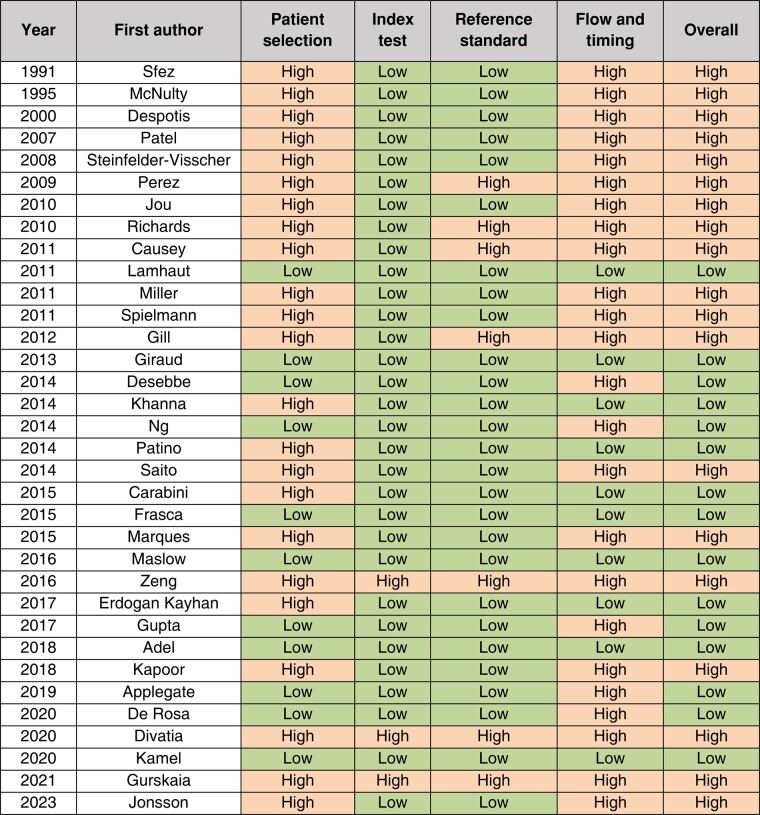

### Meta-analysis

*Table 2* shows the pooled analysis of each class of POCT device and its subgroup analyses. If the included studies had insufficient data to perform subgroup analyses, they were not reported.

**Table 2:** Meta-analysis of POCT devices and subgroups

Analysis group	Number of studies	Number of patients	Bias (g/l)	Standard deviation of bias	Between-study heterogeneity in bias (*I*^2^)	95% LOA (g/l)
Pulse Co-Ox	25	1110	2.3	1.15	0.57	(−25.2,29.8)
Pulse Co-Ox (funded)	8	423	2.1	1.17	0.98	(−31.0,35.1)
Pulse Co-Ox (unfunded)	17	687	2.2	1.14	0.18	(−22.1,26.6)
Pulse Co-Ox (blood loss > 1 litre)	5	305	−2.5	0.90	0.80	(−27.9,22.9)
HemoCue	10	525	−0.3	0.48	0.06	(−11.1,10.5)
HemoCue (arterial)	5	314	0.1	0.41	0.07	(−9.6,9.87)
HemoCue (capillary)	5	228	1.4	0.93	0.12	(−18.5,21.2)
HemoCue (venous)	2	90	−1.2	0.51	0.19	(−14.5,12.1)
iSTAT	6	146	−0.3	0.32	0.06	(−8.4,7.8)
Blood gas analyser	10	821	−2.6	0.51	0.32	(−17.8,12.7)
Low risk of bias (Pulse Co-Ox)	13	675	1.9	1.20	0.97	(−29.2,32.9)

POCT, point-of-care tests; LOA, limits of agreement.

#### Pulse co-oximeter

**Fig. 2:**
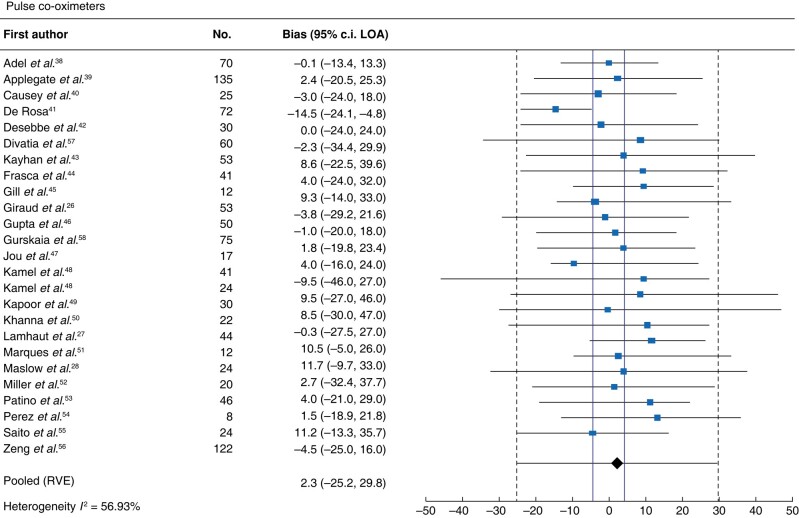
Forest plot of pulse co-oximeters Solid vertical red lines indicate allowable difference of ±4 g/l defined by the Institute of Quality Management in Healthcare. Haemoglobin units are g/l. LOA, limits of agreement. RVE, robust variance estimation.

Meta-analysis of 25 studies assessing pulse co-oximeters revealed an MD (95% LOA) of 2.3 (−25.2 to 29.8) g/l and high heterogeneity (*I*^2^ = 57%; *Fig. 2*). When subgroup analyses were performed for industry funding (*n* = 8), non-industry funding (*n* = 15) or blood loss greater than 1 litre (*n* = 5), the MDs (95% LOA) were 2.1 (−31.0 to 35.1) g/l, 2.6 (−25.0 to 30.1) g/l, and −2.5 (−27.9 to 22.9) g/l, respectively. Two studies reported MDs within the critical transfusion zone of 60–100 g/l; thus, a subgroup was not performed. Sensitivity analysis of pulse co-oximetry studies at low risk of bias (*n* = 12) revealed an MD (95% LOA) of 1.9 (−29.2 to 32.9) g/l (*Fig. S1*).

#### HemoCue

**Fig. 3:**
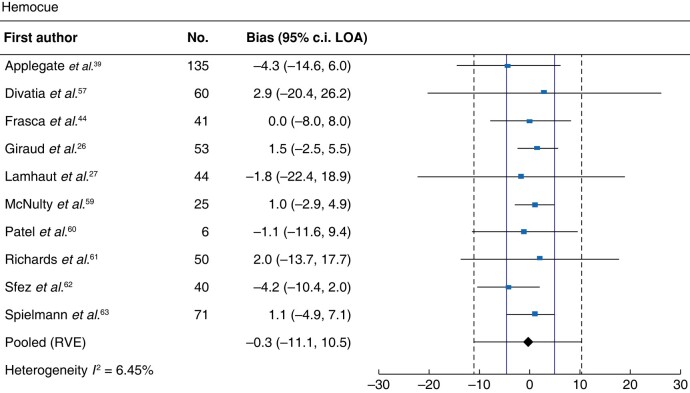
Forest plot of HemoCue Solid vertical red lines indicate allowable difference of ±4 g/l defined by the Institute of Quality Management in Healthcare. Haemoglobin units are g/l. LOA, limits of agreement. RVE, robust variance estimation.

Meta-analysis of 10 studies assessing HemoCue revealed an MD (95% LOA) of −0.3 (−11.1 to 10.5) g/l and low heterogeneity (*I*^2^ = 6%; *Fig. 3*). Subgroup analyses of arterial, capillary and venous samples demonstrated MDs (95% LOA) of 0.1 (−9.6 to 9.8) g/l, 1.4 (−18.5 to 21.2) g/l and −1.2 (−14.5 to 12.1) g/l, respectively.

#### iSTAT

**Fig. 4:**
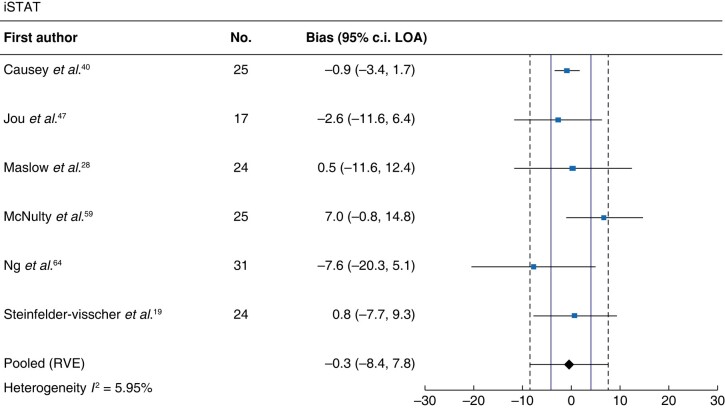
Forest plot of iSTAT Solid vertical red lines indicate allowable difference of ±4 g/l defined by the Institute of Quality Management in Healthcare. Haemoglobin units are g/l. LOA, limits of agreement. RVE, robust variance estimation.

Meta-analysis of six studies assessing iSTAT revealed an MD (95% LOA) of −0.3 (−8.4 to 7.8) g/l and low heterogeneity (*I*^2^ = 6%; *Fig. 4*).

#### Blood gas analysers

Meta-analysis of 10 studies assessing blood gas analysers revealed an MD (95% LOA) of −2.6 (−17.8 to 12.7) g/l and low heterogeneity (*I*^2^ = 32%; *Fig. S2*).

## Discussion

In this systematic review, the accuracy of point-of-care haemoglobin devices compared to central laboratory measurements in patients undergoing surgery was assessed. Most studies compared pulse co-oximetry to central laboratory measurements, with fewer studies examining HemoCue, iSTAT and blood gas analysers. Of the 34 included studies, none examined occlusion spectroscopy or transcutaneous reflection spectroscopy. Almost a third of studies were industry-funded and only a minority quantified blood loss. Only three studies compared devices within the critical transfusion zone of 60–100 g/l. Overall, the accuracies of the reported devices are inadequate to guide intraoperative transfusion decision-making. The sample size and number of available studies for each POCT device were low and further high-quality prospective accuracy studies are warranted.

To the authors' knowledge, this is the first systematic review and meta-analysis that investigates method comparison studies of POCT devices *versus* central laboratory haemoglobin measurements specifically in the operative setting. Given that haemoglobin values play a central role in transfusion decision-making in surgery, it is imperative to evaluate the accuracy of POCT devices as they are increasingly used *in lieu* of formal central laboratory assays. The current analyses demonstrate that the pooled bias (g/l) of pulse co-oximetry, HemoCue, iSTAT and blood gas analysers was 2.3, −0.3, −0.3 and −2.6, respectively. The bias indicates the difference of the mean error above and below the reference measurement but does not report the magnitude of the error in each direction. Thus, clinically, the bias alone would be insufficient to compare methods. Rather, the 95% LOAs provide the interval within which 95% of the differences between haemoglobin measurements by the two methods are expected to lie^68,69^. In this context, the 95% LOA is more clinically relevant to assess agreement between methods. The pooled 95% LOA (g/l) for pulse co-oximeters, HemoCue, iSTAT and blood gas analysers were −25.2 to 29.8, −11.1 to 10.5, −8.4 to 7.8 and −17.8 to 12.7, respectively. These intervals are much larger than the allowable difference of ±4 g/l defined by the Institute of Quality Management in Healthcare (IQMH)^70^. Alternatively, when using an allowable difference of ±10 g/l, as previously argued by Morey and colleagues^24^, the 95% LOA of iSTAT falls within this range; however, only six studies (*n* = 146 patients) were included in this meta-analysis, of which four had high risks of bias. As such, haemoglobin values measured by POCT devices should not be considered interchangeable with central laboratory values and abundant caution is needed when using these devices to guide transfusion decisions in the operating room.

Other systematic reviews and meta analyses of the accuracy of POCT devices measuring haemoglobin have been reported^21,37,71,72^, but all have significant differences compared to this review. Shabaninejad and colleagues^71^ included 28 studies comparing Radical-7 pulse co-oximetry to central laboratory measurements in the operative setting and demonstrated a bias (95% LOA) of 2.7 (−4.4 to −1.0) g/l. However, their meta-analysis included other POCT devices such as iSTAT and blood gas analysers as reference measurements. The accuracy of these devices has not been validated for use in surgery and therefore these were not considered an acceptable reference comparator in the current review. Further, Shabaninejad et al.’s review was limited to Radical-7 pulse co-oximetry, and sensitivity analyses based on study quality and risk of bias were not presented. In 2020, Zortea and colleagues^72^ included eight studies comparing haemoglobin values measured by non-invasive techniques *versus* central laboratory. Their sensitivity analysis of surgical patients in four studies revealed a mean overall difference of 0.02 (95% c.i. −0.43 to 0.47). However, this group included patients outside of the operative setting and used POCT devices such as blood gas analysers as reference standards. In 2015, Hiscock and colleagues^21^ published a meta-analysis of 39 studies comparing pulse co-oximetry and HemoCue to central laboratory haemoglobin measurements and reported a bias (95% LOA) of −0.3 (−30 to 29 g/l) and 0.8 (−13 to 14 g/l), respectively. Their results demonstrate that Masimo pulse co-oximetry devices have lower precision and wider 95% LOA compared to HemoCue, which is consistent with the current analysis; however, their review included primarily non-operative data. Further, Kim and colleagues^37^ analysed 32 studies comparing non-invasive haemoglobin measurements (Masimo, OrSense) to central laboratory testing. More specifically, a subgroup analysis of 13 studies conducted in the perioperative setting demonstrated a bias (95% LOA) of 3.9 (−22.1 to 29.8 g/l). Again, this subgroup included patients outside of the operative setting. Lastly, none of the other reviews addressed within-individual correlation between successive measurements. In the current paper, six studies reported repeated measures. For the remaining 26 studies, the standard deviation was adjusted to provide a robust variance estimation.

The current review has several limitations. Results should be interpreted carefully as 18 of 34 studies were assessed to have high overall risk of bias. It is also noteworthy that 11 studies were funded by device manufacturers. The accuracy of the POCT device may be overestimated as industry-funded studies that show a larger difference between the POCT device and the central laboratory may be less likely to be published. In addition, a high level of heterogeneity was identified in the pulse co-oximetry group, likely owing in part to different operation types, blood loss, and intraoperative interventions such as cardiopulmonary bypass and acute normovolaemic haemodilution, which may affect haemoglobin measurements. Only three studies reported blood transfusions^41,46,55^. In the study of De Rosa, transfusion was performed if haemoglobin was <80 g/l in healthy patients or <90 g/l in patients with cardiac disease or active bleeding. In Gupta *et al*., blood transfusion was at the discretion of the anaesthesiologist and parameters were not specified. No patients received allogeneic blood transfusions in Saito *et al*.

Intraoperative decision-making for RBC transfusions is complex and is not based on a robust evidence base. A 2021 systematic review of clinical practice guidelines for intraoperative RBC transfusions identified 10 guidelines^73^. However, recommendations were highly variable and data were extrapolated from non-operative settings. Further, none provided recommendations on the most appropriate method for haemoglobin measurement, although all implied that transfusions should be guided at least in part by haemoglobin/haematocrit triggers, thresholds and/or targets. No guideline discussed the role of point-of-care haemoglobin testing.

This review suggests that POCT haemoglobin devices are insufficiently accurate to be used interchangeably with central laboratory haemoglobin testing in the operating room. This finding is particularly important as it pertains to transfusion decision-making, and inaccurate haemoglobin measurements could lead to over- or under-transfusion, both of which can lead to significant patient harm. Further prospective accuracy data are required to compare the accuracy of POCT haemoglobin devices in the operative setting, principally within the critical transfusion zone of 60–100 g/l, as well as to determine their ability to appropriately guide transfusion.

## Supplementary Material

zrad148_Supplementary_Data

## Data Availability

The authors declare that the data supporting the findings of this study are available within the article and its supplementary files.
