# Supplementary material for: Accuracy of point-of-care testing devices for haemoglobin in the operating room: meta-analysis
Source: BJS Open. 2024 Jan 24;8(1):zrad148. doi: 10.1093/bjsopen/zrad148 (PMC10807999; doi:10.1093/bjsopen/zrad148)
Supplement: zrad148_Supplementary_Data [file zrad148_supplementary_data.docx]

**Accuracy of point of care testing devices for haemoglobin in the operating room: meta-analysis**

Hilalion (San) Ahn,^1^ Tori Lenet,^1,2^ Richard WD Gilbert,^1^ Ranjeeta Mallick,^2^ Julie LV Shaw,^3^ Dean A Fergusson,^2^ Daniel I McIsaac,^2,4^ Guillaume Martel,^1,2^

^1^Department of Surgery, The Ottawa Hospital, University of Ottawa, Ottawa, ON

^2^Clinical Epidemiology Program, Ottawa Hospital Research Institute, Ottawa, ON

^3^Department of Pathology and Laboratory Medicine, University of Ottawa, Ottawa, ON

^4^Departments of Anesthesiology & Pain Medicine, The Ottawa Hospital, University of Ottawa, Ottawa, ON

**Corresponding author.**

Dr. Guillaume Martel

The Ottawa Hospital, General Campus

501 Smyth Road, CCW 1667, Ottawa, ON, Canada, K1H 8L6

**ORCID ID 0000-0002-2199-3772**; **Twitter @ChamoGui**

**Supplementary Materials - Index**

| **Supplementary Methods** |  |
| --- | --- |
| Search strategy | *pag. 2* |
| QUADAS-2 Risk of bias questionnaire | *pag. 4* |
| **Supplementary Results** |  |
| Figure S1. Subgroup analysis of Pulse Co-oximetry low risk of bias studies | *pag. 6* |
| Figure S2. Forest plot of blood gas analyzers | *pag. 7* |
| **Supplementary Figures and Tables** |  |
| Table S1. PRISMA checklist | *pag. 8* |
| Table S2. Study characteristics | *pag. 10* |
| **References** | *pag. 13* |
|  |  |

**Supplementary Methods**

Search strategy

Embase Classic+Embase <1947 to 2023 August 18>

Ovid MEDLINE(R) ALL <1946 to August 18, 2023>

EBM Reviews - Cochrane Central Register of Controlled Trials <July 2023>

**Medline**

1 Point-of-Care Systems/ 21102

2 Point-of-Care Testing/ 25419

3 (point of care test* or poct).tw,kw. 22103

4 oximetry/mt 3684

5 (pulse adj5 co oximet*).tw. or pulse co oximetry.kw. 586

6 (Masimo or Radical-7 or Rad-57 or Rad-67 or Rad-97 or Radius-7 or Pronto-7).tw,kw. 1554

7 OrSense.tw,kw. 59

8 Haemospect.tw,kw. 32

9 noninvasive spectroscop*.tw,kw. 64

10 transcutaneous reflection spectroscopy.tw,kw. 0

11 Occlusion spectroscop*.tw,kw. 36

12 HemoCue.tw,kw. 1777

13 DiaSpect.tw,kw. 16

14 (i-STAT or iSTAT).tw,kw. 1962

15 epoch.tw,kw. 14588

16 or/1-15 79055

17 Hemoglobins/ 317628

18 (H?emoglobin* or hb or hbg).tw,kw. 614331

19 Hemoglobinometry/ 36213

20 17 or 18 or 19 740917

21 16 and 20 4910

22 ((noninvasive or non invasive or bedside) adj3 (h?emoglobin* or hgb)).tw. 773

23 (noninvasive h?emoglobin* or non invasive h?emoglobin* or noninvasive hgb or non invasive hgb).kw. 69

24 or/21-23 5252

25 (measur* or monitor* or accuracy).mp. 13888222

26 24 and 25 3984

27 exp animals/ not humans/ 17612892

28 26 not 27 3130

**29** **28 use medall** **1448**

**30** **limit 29 to dt=20200424-20230821** **254**

**Embase**

31 "point of care testing"/ 25419

32 (point of care test* or poct).tw. 19447

33 "point of care system"/ 20575

34 co oximet*.tw. 1963

35 (Masimo or Radical-7 or Rad-57 or Rad-67 or Rad-97 or Radius-7 or Pronto-7).tw. 1549

36 OrSense.tw. 59

37 Haemospect.tw. 32

38 noninvasive spectroscop*.tw. 64

39 transcutaneous reflection spectroscopy.tw. 0

40 Occlusion spectroscop*.tw. 36

41 HemoCue.tw. 1769

42 DiaSpect.tw. 16

43 (i-STAT or iSTAT).tw. 1946

44 epoch.tw. 14569

45 *pulse oximeter/ 671

46 31 or 32 or 33 or 34 or 35 or 36 or 37 or 38 or 39 or 40 or 41 or 42 or 43 or 44 or 45 75496

47 hemoglobin/ or hemoglobin determination/ 358815

48 (H?emoglobin* or hb or hbg).tw. 604351

49 Hemoglobinometr*.tw. 218

50 47 or 48 or 49 739405

51 46 and 50 4931

52 ((noninvasive or non invasive or bedside) adj3 (h?emoglobin* or hgb)).tw. 773

53 51 or 52 5268

54 (monitor* or measur* or accuracy).mp. 13888222

55 53 and 54 3961

56 (exp animals/ or animal experiment/) not exp humans/ 11399146

57 55 not 56 3719

**58** **57 use emczd** **2331**

**59** **limit 58 to dc=20200427-20230821** **561**

**Cochrane**

60 Point-of-Care Systems/ 21102

61 Point-of-Care Testing/ 25419

62 (point of care test* or poct).tw,kw. 22103

63 oximetry/mt 3684

64 (pulse adj5 co oximet*).tw. or pulse co oximetry.kw. 586

65 (Masimo or Radical-7 or Rad-57 or Rad-67 or Rad-97 or Radius-7 or Pronto-7).tw,kw. 1554

66 OrSense.tw,kw. 59

67 Haemospect.tw,kw. 32

68 noninvasive spectroscop*.tw,kw. 64

69 transcutaneous reflection spectroscopy.tw,kw. 0

70 Occlusion spectroscop*.tw,kw. 36

71 HemoCue.tw,kw. 1777

72 DiaSpect.tw,kw. 16

73 (i-STAT or iSTAT).tw,kw. 1962

74 epoch.tw,kw. 14588

75 or/60-74 79055

76 Hemoglobins/ 317628

77 (H?emoglobin* or hb or hbg).tw,kw. 614331

78 Hemoglobinometry/ 36213

79 76 or 77 or 78 740917

80 75 and 79 4910

81 ((noninvasive or non invasive or bedside) adj3 (h?emoglobin* or hgb)).tw. 773

82 (noninvasive h?emoglobin* or non invasive h?emoglobin* or noninvasive hgb or non invasive hgb).kw. 69

83 or/80-82 5252

84 (measur* or monitor* or accuracy).mp. 13888222

85 83 and 84 3984

86 85 use cctr 230

87 limit 86 to yr="2020 -Current" 67

88 30 or 59 or 87 882

89 remove duplicates from 88 680

QUADAS-2 Risk of bias questionnaire

| **ASSESSMENT OF RISK OF BIAS (QUALITY CRITERIA)** | | | | | | | | | | |
| --- | --- | --- | --- | --- | --- | --- | --- | --- | --- | --- |
| Article number |  | Author |  | Year |  | Date |  | Reviewer |  | |
| Risk of bias is judged as “low,” “high,” or “unclear.” If the answers to all signaling questions for a domain are “yes,” then risk of bias can be judged low. If any signaling question is answered “no,” potential for bias exists. The “unclear” category should be used only when insufficient data are reported to permit a judgment.  Overall, if there are two or more domains in which the Risk of Bias is deemed “high”, the overall judgement would be deemed “high” risk of bias. | | | | | | | | | | |
| **Patient selection** | | | | | | | | | | |
| *Risk of Bias: Could the Selection of Patients Have Introduced Bias? (Unclear: ≥2 unclear, 1 No + 1 unclear) (High: ≥2 No)* | | | | | | | | *Low* | *High* | *Unclear* |
| Signaling question 1: Were subject population of interest specified? | | | | | | | | Yes | No | Unclear |
| Signaling question 2: Were the sampling method (e.g., at random, consecutive, convenient) for subjects stated? | | | | | | | | Yes | No | Unclear |
| Signaling question 3: Was *a priori* calculation of the number of patients needed explained according to unique hypothesis (primary end-point) being tested? | | | | | | | | Yes | No | Unclear |
| Signaling question 4: Was the population demographic data well reported in the Results? | | | | | | | | Yes | No | Unclear |
| *Applicability: Are There Concerns That the Included Patients and Setting Do Not Match the Review Question?* | | | | | | | | *Low* | *High* | *Unclear* |
| **Index test (Point of Care Hemoglobin Testing)** | | | | | | | |  |  |  |
| *Risk of Bias: Could the Conduct or Interpretation of Point of Care Hemoglobin Testing Have Introduced Bias? (2 or more No / 1 No and 1 Unclear = High)* | | | | | | | | *Low* | *High* | *Unclear* |
| Signaling question 1: Were point of care hemoglobin measurements of interest described explicitly? (i.e., Name of device, software version, or version of sensor software) | | | | | | | | Yes | No | Unclear |
| Signaling question 2: Were point of care hemoglobin measurements process clearly described to be replicated by other investigator? (i.e., measurement site, protection from ambient light, followed manufacturer instruction etc.) | | | | | | | | Yes | No | Unclear |
| *Applicability: Are There Concerns That the Point of Care Hemoglobin Monitoring, Its Conduct, or Its Interpretation Differ From the Review Question?* | | | | | | | | *Low* | *High* | *Unclear* |
| **Reference standard (Central laboratory Hemoglobin measurement)** | | | | | | | |  |  |  |
| *Risk of Bias: Could the Central laboratory Hemoglobin Measurement, Its Conduct, or Its Interpretation Have Introduced Bias? (2 or more No / 1 No and 1 Unclear = High)* | | | | | | | | *Low* | *High* | *Unclear* |
| Signaling question 1: Was the central laboratory hemoglobin measurement likely to correctly measure hemoglobin? | | | | | | | | Yes | No | Unclear |
| Signaling question 2: Were the device name and company of central laboratory device used in the reference hemoglobin measurement clearly described? | | | | | | | | Yes | No | Unclear |
| *Applicability: Are There Concerns That the Target Condition as Defined by the Central laboratory Hemoglobin Monitoring Does Not Match the Question?* | | | | | | | | *Low* | *High* | *Unclear* |
| **Flow and Timing** | | | | | | | |  |  |  |
| *Risk of Bias: Could the analysis of Flow and Timing Have Introduced Bias?*  *(Unclear: ≥2 unclear, 1 No + 1 unclear) (High: ≥2 No), Not applicable = Yes* | | | | | | | | *Low* | *High* | *Unclear* |
| Signaling question 1: Was the type of study stated (superiority, equivalent, inferiority)? | | | | | | | | Yes | No | Unclear |
| Signaling question 2: Were the statistical plan decided *a Priori*? | | | | | | | | Yes | No | Unclear |
| Signaling question 3: In case of study performing repeated measurements in same patient, did they use statistical analysis for agreement between methods of measurement with multiple observations per individual? | | | | | | | | Yes | No | Unclear |
| Signaling question 4: Was the interval between point of care and laboratory hemoglobin measurement appropriate for the study purpose (continuous *vs* point-of-care) and the method of acquiring paired measurements well described? | | | | | | | | Yes | No | Unclear |
| Signaling question 5: Were number of patients enrolled and who dropped out clearly described in the result? | | | | | | | | Yes | No | Unclear |
| Signaling question 6: In the case of the bias being described both in text and figures, do they match consistently? | | | | | | | | Yes | No | Not applicable |

**Supplementary Results**

Figure S1. Subgroup analysis of pulse co-oximetry low risk of bias studies

**
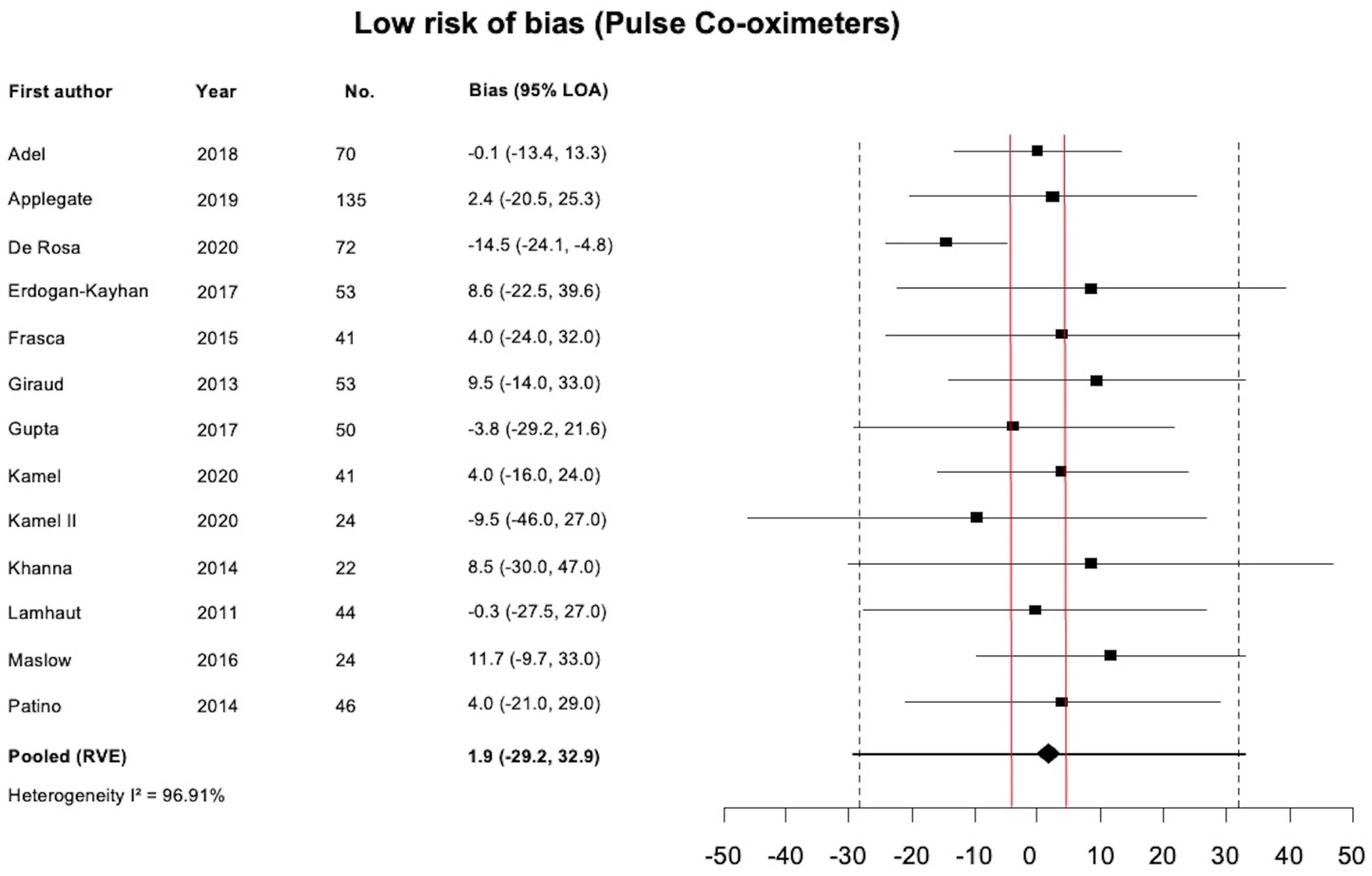
**

Solid vertical red lines indicate allowable difference of +/-4 g/L defined by the Institute of Quality Management in Healthcare. Haemoglobin units are g/L. LOA = limits of agreement. RVE = robust variance estimation.

Figure S2. Forest plot of blood gas analyzers


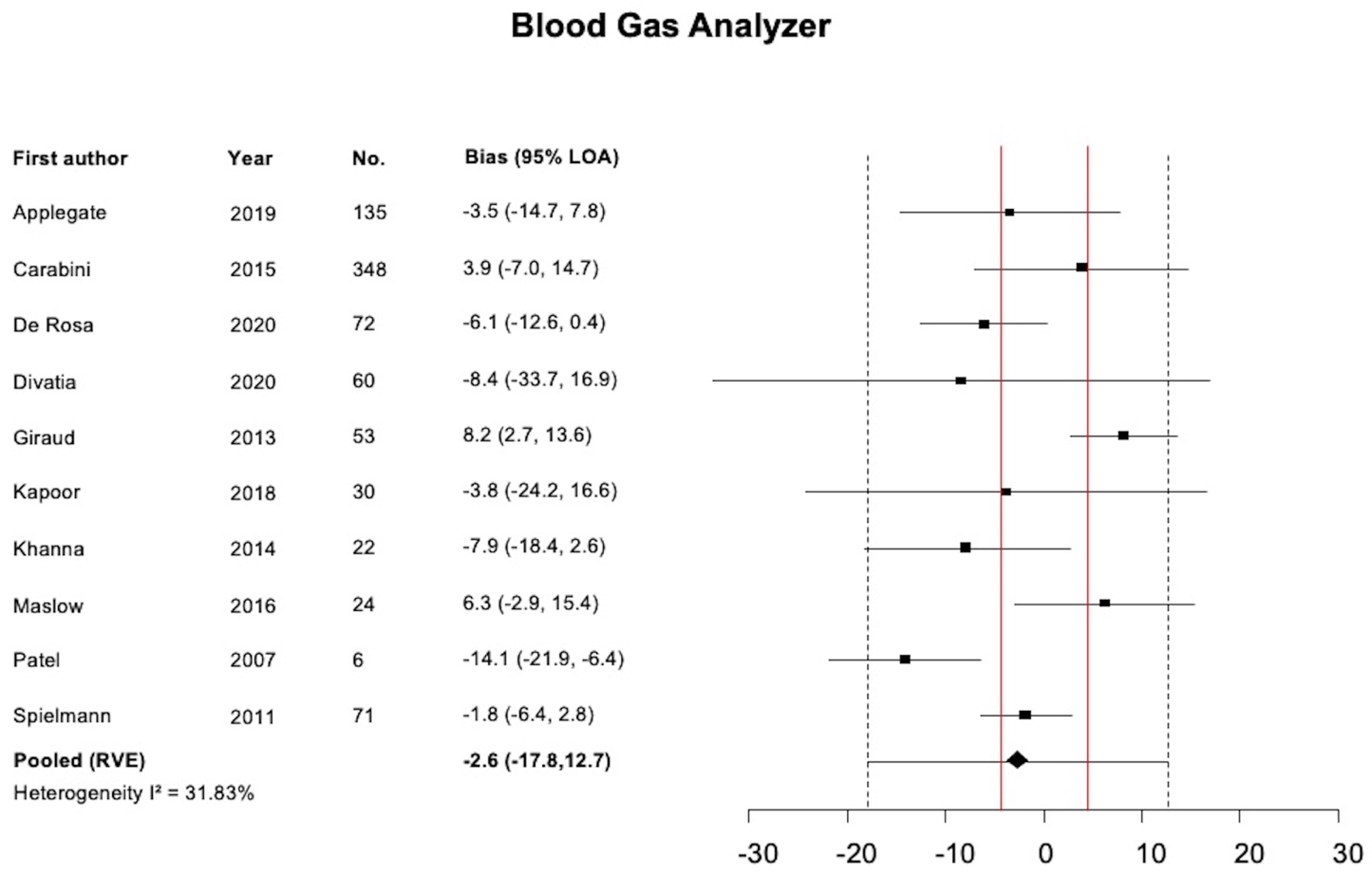


Solid vertical red lines indicate allowable difference of +/-4 g/L defined by the Institute of Quality Management in Healthcare. Haemoglobin units are g/L. LOA = limits of agreement. RVE = robust variance estimation.

**Supplementary Figures and Tables**

Table S1. PRISMA checklist

| **Section and Topic** | **Item #** | **Checklist item** | **Location where item is reported** |
| --- | --- | --- | --- |
| **TITLE** | | |  |
| Title | 1 | Identify the report as a systematic review. | 1 |
| **ABSTRACT** | | |  |
| Abstract | 2 | See the PRISMA 2020 for Abstracts checklist. | 3 |
| **INTRODUCTION** | | |  |
| Rationale | 3 | Describe the rationale for the review in the context of existing knowledge. | 4-5 |
| Objectives | 4 | Provide an explicit statement of the objective(s) or question(s) the review addresses. | 5 |
| **METHODS** | | |  |
| Eligibility criteria | 5 | Specify the inclusion and exclusion criteria for the review and how studies were grouped for the syntheses. | 6-7 |
| Information sources | 6 | Specify all databases, registers, websites, organisations, reference lists and other sources searched or consulted to identify studies. Specify the date when each source was last searched or consulted. | 6 |
| Search strategy | 7 | Present the full search strategies for all databases, registers and websites, including any filters and limits used. | 6, S2 |
| Selection process | 8 | Specify the methods used to decide whether a study met the inclusion criteria of the review, including how many reviewers screened each record and each report retrieved, whether they worked independently, and if applicable, details of automation tools used in the process. | 7-8 |
| Data collection process | 9 | Specify the methods used to collect data from reports, including how many reviewers collected data from each report, whether they worked independently, any processes for obtaining or confirming data from study investigators, and if applicable, details of automation tools used in the process. | 7-8 |
| Data items | 10a | List and define all outcomes for which data were sought. Specify whether all results that were compatible with each outcome domain in each study were sought (e.g. for all measures, time points, analyses), and if not, the methods used to decide which results to collect. | 8 |
|  | 10b | List and define all other variables for which data were sought (e.g. participant and intervention characteristics, funding sources). Describe any assumptions made about any missing or unclear information. | 8 |
| Study risk of bias assessment | 11 | Specify the methods used to assess risk of bias in the included studies, including details of the tool(s) used, how many reviewers assessed each study and whether they worked independently, and if applicable, details of automation tools used in the process. | 9 |
| Effect measures | 12 | Specify for each outcome the effect measure(s) (e.g. risk ratio, mean difference) used in the synthesis or presentation of results. | 8-9 |
| Synthesis methods | 13a | Describe the processes used to decide which studies were eligible for each synthesis (e.g. tabulating the study intervention characteristics and comparing against the planned groups for each synthesis (item #5)). | 8-9 |
|  | 13b | Describe any methods required to prepare the data for presentation or synthesis, such as handling of missing summary statistics, or data conversions. | 8-9 |
|  | 13c | Describe any methods used to tabulate or visually display results of individual studies and syntheses. | 8-9 |
|  | 13d | Describe any methods used to synthesize results and provide a rationale for the choice(s). If meta-analysis was performed, describe the model(s), method(s) to identify the presence and extent of statistical heterogeneity, and software package(s) used. | 8-9 |
|  | 13e | Describe any methods used to explore possible causes of heterogeneity among study results (e.g. subgroup analysis, meta-regression). | 8-9 |
|  | 13f | Describe any sensitivity analyses conducted to assess robustness of the synthesized results. | 8-9 |
| Reporting bias assessment | 14 | Describe any methods used to assess risk of bias due to missing results in a synthesis (arising from reporting biases). | 8-9 |
| Certainty assessment | 15 | Describe any methods used to assess certainty (or confidence) in the body of evidence for an outcome. | 8-9 |
| **RESULTS** | | |  |
| Study selection | 16a | Describe the results of the search and selection process, from the number of records identified in the search to the number of studies included in the review, ideally using a flow diagram. | 10, Fig 1 |
|  | 16b | Cite studies that might appear to meet the inclusion criteria, but which were excluded, and explain why they were excluded. | 10-11, Fig 1 |
| Study characteristics | 17 | Cite each included study and present its characteristics. | 10-11, Table 1 |
| Risk of bias in studies | 18 | Present assessments of risk of bias for each included study. | 11, Table 2 |
| Results of individual studies | 19 | For all outcomes, present, for each study: (a) summary statistics for each group (where appropriate) and (b) an effect estimate and its precision (e.g. confidence/credible interval), ideally using structured tables or plots. | 12 |
| Results of syntheses | 20a | For each synthesis, briefly summarise the characteristics and risk of bias among contributing studies. | 12-13, Fig 2-4 |
|  | 20b | Present results of all statistical syntheses conducted. If meta-analysis was done, present for each the summary estimate and its precision (e.g. confidence/credible interval) and measures of statistical heterogeneity. If comparing groups, describe the direction of the effect. | 12-13, Fig 2-4 |
|  | 20c | Present results of all investigations of possible causes of heterogeneity among study results. | 12 |
|  | 20d | Present results of all sensitivity analyses conducted to assess the robustness of the synthesized results. | 12 |
| Reporting biases | 21 | Present assessments of risk of bias due to missing results (arising from reporting biases) for each synthesis assessed. | 12 |
| Certainty of evidence | 22 | Present assessments of certainty (or confidence) in the body of evidence for each outcome assessed. | 12-13 |
| **DISCUSSION** | | |  |
| Discussion | 23a | Provide a general interpretation of the results in the context of other evidence. | 13-14 |
|  | 23b | Discuss any limitations of the evidence included in the review. | 15 |
|  | 23c | Discuss any limitations of the review processes used. | 15 |
|  | 23d | Discuss implications of the results for practice, policy, and future research. | 15 |
| **OTHER INFORMATION** | | |  |
| Registration and protocol | 24a | Provide registration information for the review, including register name and registration number, or state that the review was not registered. | 6 |
|  | 24b | Indicate where the review protocol can be accessed, or state that a protocol was not prepared. | 6 |
|  | 24c | Describe and explain any amendments to information provided at registration or in the protocol. | 6 |
| Support | 25 | Describe sources of financial or non-financial support for the review, and the role of the funders or sponsors in the review. | 1 |
| Competing interests | 26 | Declare any competing interests of review authors. | 1 |
| Availability of data, code and other materials | 27 | Report which of the following are publicly available and where they can be found: template data collection forms; data extracted from included studies; data used for all analyses; analytic code; any other materials used in the review. | 7-10 |

Table S2. Study characteristics

| **First Author** | **Year** | **Type of Surgery** | **Funding source** | **Age** mean (SD), *median [range]* | **Sex Ratio (M:F)** | **Sample Size** | **# of Paired Measurements** | **Laboratory Analyzer** | **Test Device(s)**  (software version, sensor version) | **Mean Difference** (device-lab) (g/L) | **95% LOA** (g/L) |
| --- | --- | --- | --- | --- | --- | --- | --- | --- | --- | --- | --- |
| Sfez | 1991 | - | Not reported | - | - | 40 | 47 | STKS | Capillary HemoCue Venous HemoCue | -2.72 -1.66 | -23.56;18.12 -10.41;1.95 |
| McNulty | 1995 | Cardiac | Not reported | - | - | 25 | 90 | STKR | Nova Stat Profile 9 HemoCue | 7.0 1.0 | -1.0;15.0 -3.0;5.0 |
| Despotis | 2000 | Cardiac requiring CPB | Not reported | - | - | 204 | 408 | STKS | Beckman-Coulter T540 | -0.03 | -4.93;4.87 |
| Patel | 2007 | Cardiac requiring CPB | None | - | - | 6 | 68 | LH750 | HemoCue Hb 201+ Rapidpoint 405 IL 682 | -1.1 -0.015 1.37 | -11.58;9.37 -4.11;4.08 -3.28;6.02 |
| Steinfelder-Visscher | 2008 | Cardiac requiring CPB | None | - | - | 48 | 24 | Sysmex XE-2100 | iSTAT | 1.0 | -7.7;9.3 |
| Perez | 2009 | Orthopaedic, abdominal | Not reported | 68.6 (24.7) | - | 8 | 15 | - | Masimo Pulse Co-Oximeter | 1.45 | -18.9;21.8 |
| Jou | 2010 | Craniotomy, cardiac, spinal, liver transplant | Not reported | 9.3 (5.9) | 23.5:76.5 | 17 | 92 45 | Cell-Dyn Sapphire | Rainbow SET Pulse Co-Oximeter (7.4.09/7.5.03, R120-L/R125-L Rev C) iSTAT | 1.8 -2.6 | -19.8;23.4 -11.6;6.4 |
| Richards | 2010 | Elective c-section | HemoCue | - | 0 | 50 | 50 | - | Capillary HemoCue Venous HemoCue | 1.0  2.0 | -17;19 -14;18 |
| Causey | 2011 | Elective major | Not reported | - | - | 25 | 101 27 | - | Radical-7 (7.5.0.3, R125 Rev C) iStat | -2.9 -0.9 | -24;18 NR |
| Lamhaut | 2011 | Major urologic surgery | Masimo | 58 (13) | 70.5:29.5 | 44 | 85 | Sysmex SP-1000i | Radical-7 (7.4.09, Adhesive Rev C) HemoCue (201+) | -0.2 -1.7 | -27.5; 27 -22.4;18.9 |
| Miller | 2011 | Spine | Masimo, University of California San Francisco | - | - | 20 | 78 | Beckman-Coulter | Radical-7 (Adhesive Rev E) HemoCue | 2.6 NR | -32;38 NR |
| Spielmann | 2011 | Elective surgery with major EBL (craniofacial, spine, hip, cancer) | None | *3.3 [NR]* | - | 71 | 50 | Sysmex XE-2100 | HemoCue (B-Hemoglobin) GEM OPL | 1.1 -1.8 | -4.9;7.1 -6.4;2.8 |
| Gill | 2012 | Craniofacial | Masimo | - | - | 12 | 45 | - | Masimo Pulse Co-Oximeter | -9.3 | -28.5;9.9 |
| Giraud | 2013 | Major surgery with significant expected blood loss | Masimo, University hospital of Poitiers | *63 [55-74]* | 57:43 | 53 | 219 | Sysmex XT-2100i | Radical-7 (7.6.0.1, R2-25 Rev E) Capillary HemoCue (Hb201)  Arterial HemoCue (Hb201) RapidPoint 405 | 9.5 5.4 1.5 8.2 | -14.0;33.0 -5.6;15.6 -2.5;5.5 2.7;13.6 |
| Desebbe | 2014 | Cardiac | Not reported | *68 [29-89]* | - | 30 | 90 | - | FORE-SIGHT cerebral oximeter | 0 | -24;24 |
| Khanna | 2014 | Neurosurgery | None | 30.19 (11.63) | 54.5:45.5 | 22 | 22 | Automated hemoglobin analyzer | Radical-7 ABG | 10 -8.0 | -30;47 -18.4;2.6 |
| Ng | 2014 | Any procedure with EBL >25% | Abbott (iSTAT) | 62 [14-88] | 51.6:48.4 | 31 | 30 | Sysmex XE-2100 | iSTAT (CG8+ cartridges) | -5.1 | -291;189 |
| Patino | 2014 | Major surgical procedures with substantial blood loss (cardiac, liver, spine, craniofacial) | Masimo | Pediatric | 63:37 | 46 | 140 | Cell-Dyn Sapphire | Radical-7 (7.6.21/7.8.01, R1-20L/R1-25L Rev E or R2-20 or R2-25 Rev E) | 4.0 | -21;29 |
| Saito | 2014 | Urological and gynaecological surgery | None | 61 (12) | 71:29 | 24 | 228 | Sysmex KX-21N | Radical-7 (7.6.01, ReSposable) | 11.2 | -13.8;37 |
| Carabini | 2015 | Spinal fusion >3 bony levels | Northwestern University | - | - | 348 | 1832 | LH750 | GEM Premier 4000 | 3.9 | -7.0;14.7 |
| Frasca | 2015 | Elective major surgery with significant expected blood loss | Masimo, University hospital of Poitiers | *65 [34-83]* | 29:71 | 41 | 173 | Sysmex XT-2100i | Radical-7 (7.8.0.1, R2–25 Rev G)  Capillary HemoCue (Hb201)  Arterial HemoCue (Hb201) | 4.0 -5.0 0 | -24;32 -2.9;19 -8;8 |
| Marques | 2015 | Controlled haemorrhage with haemodilution | Office of Naval Research | 28 (3) | 66.7:33.3 | 12 | 106 | Sysmex XE-2100/XT-1800 | Radical-7 (R2-25r) | 10.8 | -5.0;26 |
| Maslow | 2016 | Cardiac requiring CPB | Not reported | 79.9 (7) | - | 24 | 95 91 92 | UniCel DxH 800 | Radical-7 (7.6.1.1, R125/R125L) iSTAT GEM 4000 (GEM OPL co-oximeter) | 11.6 0.5 6.3 | -9.7;33 -11.6;12.5 -2.9;15.4 |
| Zeng | 2016 | General surgery | None | 4.2 (1.6) | - | 122 | 122 | - | Pulse Co-Oximeter | -4.4 | -25;16 |
| Erdogan Kayhan | 2017 | Liver transplant | None | 46.9 (12.7) | - | 53 | 282 | LH780 | Radical-7 (7.8.0.1, R2-25a/R2-25r) | 8.6 | -22.5;39.6 |
| Gupta | 2017 | Elective oncosurgery | None | *45 [18-75]* | 62:38 | 50 | 137 | Beckman-Coulter | Radical-7 | -3.76 | -29.2;21.6 |
| Adel | 2018 | Elective major orthopaedic | Cairo University | *49 [39-55]* | 43:57 | 70 | 210 | LH750 | Radical-7 | -0.1 | -13.4;13.3 |
| Kapoor | 2018 | Pituitary surgery | None | 40.83 (17.03) | 43.3:56.7 | 30 | 30 | Automated hemoglobin analyzer | Pronto Eschweiler ABG analyzer | 9.0 -3.77 | -26.96;45.96 -24.15;16.62 |
| Applegate | 2019 | Non-cardiac with arterial catheterization | Masimo | *61 [50-69]* | 48.1:51.9 | 135 | 551 | Sysmex XE-5000 or LH750 | Radical-7 (R125 Rev K) HemoCue (HB 301) ABL 800/RapidLab 1265 | 2.4 -4.3 -3.6 | -20.5;25.3 -14.6;6.0 -14.5;7.8 |
| De Rosa | 2020 | Open elective aortic abdominal surgery | Masimo | 68.8 (7.7) | 90.3:9.7 | 72 | 288 | LH750 | Radical-7 (VKF-RAD7A, R2-25a/R2-25r) GEM Premier 4000 | -14.5 -6.1 | -24.1;-4.8 -12.6;0.4 |
| Divatia | 2020 | Oncosurgery with anticipated blood loss >21mL/kg | Not reported | - | - | 60 | 183 | Siemens laboratory analyzer | Radical-7 (R-25a)  Roche COBAS-B221 co-oximeter  HemoCue | -2.3  -8.4  2.9 | -34.4;29.9  -33.7;16.9  -20.4;26.2 |
| Kamel | 2020 | Cardiac procedure (surgery or catheterization) | None | *1.5 [1.2-4]* | 39 | 65:35 | 74 37 | LH750 | Radical-7 Acyanotic Radical-7 Cyanotic | 4.0 -10 | -16;24 -46;27 |
| Gurskaia | 2021 | Craniofacial | Not reported | *[6 months – 1.5 years]* | - | 75 | 257 | - | Radical-7 | 10 | -20;18 |
| Jonsson | 2023 | Major surgery | PixCell Medical | 65 (1.5) | 60.4;39.6 | 91 | 145 | Sysmex XN-9000 | Hemoscreen | 1.1 | 7.3;9.4 |

**References**

Whiting PF, Rutjes AW, Westwood ME, Mallett S, Deeks JJ, Reitsma JB, Leeflang MM, Sterne JA, Bossuyt PM, QUADAS-2 Group*. QUADAS-2: a revised tool for the quality assessment of diagnostic accuracy studies. Annals of internal medicine. 2011 Oct 18;155(8):529-36.

Page MJ, Moher D, Bossuyt PM, Boutron I, Hoffmann TC, Mulrow CD, Shamseer L, Tetzlaff JM, Akl EA, Brennan SE, Chou R. PRISMA 2020 explanation and elaboration: updated guidance and exemplars for reporting systematic reviews. bmj. 2021 Mar 29;372.
